# Single-Cell RNA Sequencing Reveals Macrophage Dynamics During MASH in *Leptin*-Deficient Rats

**DOI:** 10.3390/cells14020096

**Published:** 2025-01-10

**Authors:** Xiaoming Xin, Yaohua Ni, Jing Wang, Fenglin Wu, Meichen Liu, Lingjuan Wu, Jiaxing Dai, Chenglin Wu, Xiaolei Song, Wang Zhang, Guangrui Yang, Ruling Shen, Xianmin Zhu

**Affiliations:** 1 School of Pharmacy, Shanghai University of Medicine and Health Sciences, Shanghai 201318, China; xmxin1687@163.com (X.X.); ericani16@outlook.com (Y.N.); wangjing02212024@126.com (J.W.); 13140728648@163.com (M.L.); lingjuanwu1@163.com (L.W.); 17873421679@163.com (J.D.); wucl@sumhs.edu.cn (C.W.); songxl@sumhs.edu.cn (X.S.); 2 School of Clinical Medicine, Shanghai University of Medicine and Health Sciences, Shanghai 201318, China; wfl050123@126.com (F.W.); yanggr@sumhs.edu.cn (G.Y.); 3 Shanghai Institute for Advanced Immunochemical Studies, ShanghaiTech University, Shanghai 201210, China; zhangwang@shanghaitech.edu.cn; 4 Shanghai Academy of Sciences & Technology Institute of Model Animals Transformation, Shanghai Laboratory Animal Research Center, Shanghai 201203, China

**Keywords:** MASH, inflammation, single cell RNA sequencing, macrophage, rat

## Abstract

Macrophages play important roles in metabolic dysfunction-associated steatohepatitis (MASH), an advanced and inflammatory stage of metabolic dysfunction-associated steatotic liver disease (MASLD). In humans and mice, the cellular heterogeneity and diverse function of hepatic macrophages in MASH have been investigated by single cell RNA sequencing (scRNA-seq). However, little is known about their roles in rats. Here, we collected liver tissues at the postnatal week 16, when our previously characterized *Lep^∆I14/∆I14^* rats developed MASH phenotypes. By scRNA-seq, we found an increase in the number of macrophages and endothelial cells and a decrease in that of NK and B cells. Hepatic macrophages in rats underwent a unique M1 to M2 transition without expression of the classical markers such as Arg1 and Nos2, except for Cd163. Lipid-associated macrophages (LAMs) were increased, which could be detected by the antibody against Cd63. In the microenvironment, macrophages had an increased number of interactions with hepatocytes, myofibroblasts, T cells, neutrophils, and dendritic cells, while their interaction strengths remained unchanged. Finally, the macrophage migration inhibitory factor (MIF) pathway was identified as the top upregulated cell-communication pathway in MASH. In conclusion, we dissected hepatic macrophage dynamics during MASH at single cell resolution and provided fundamental tools for the investigation of MASH in rat models.

## 1. Introduction

Metabolic dysfunction-associated steatotic liver disease (MASLD, previously known as nonalcoholic fatty liver disease NAFLD) is the most common liver disease worldwide, affecting about 25% of the population [1]. Metabolic dysfunction-associated steatohepatitis [MASH, or previously known as nonalcoholic steatohepatitis (NASH)] is an advanced and inflammatory stage of MAFLD [2], which further develops into liver fibrosis, cirrhosis, and hepatocellular carcinoma (HCC) [3]. More than 80% cases of MASH develop in obese patients, whose pathological features include steatosis, hepatocyte ballooning, lobular inflammation, and fibrosis [4]. After many years of failure in searching the medicine and treatment for MASH, a thyroid hormone receptor beta (THR-β) agonist (Resmetirom) has won the marathon and been approved by the US Food and Drug Administration (FDA) [5,6]. Although Resmetirom targets the metabolism of fatty acids [6], its success inspires the research fields in other pathways such as inflammation and fibrogenesis. Indeed, different cell types in the liver can be targeted for the treatment of MASH. For example, hepatocytes mainly contribute to the abnormalities in metabolism, while hepatic stem cells (HSCs) and myofibroblasts (MFs) are associated with fibrogenesis. Macrophages, the key innate immune cells, have been shown to play important roles during MASH progression [7,8,9]. However, the detailed mechanisms remain elusive.

Recent advances in single cell RNA sequencing (scRNA-seq) facilitate the characterization of cellular heterogeneity and diverse function of liver macrophages in MASH. In mice, most of the resident liver macrophages are Kupffer cells (KCs). They originate from the yolk sac or the fetal liver, which are then maintained by local proliferation [7]. The other macrophages are recruited from bone marrow-derived circulating monocytes (monocytes derived macrophages, MdMs), which are usually categorized into inflammatory monocytes (Ly6C^high^) and patrolling monocytes (Ly6C^low^). In contrast, human liver macrophages are mainly from circulating monocytes [7]. In both mice and humans, KCs are decreased and MdMs are increased in MASH. The scRNA-seq of healthy human liver tissues identified two major cell types based on CD68 and MARCO expression [10]. The CD68^+^ MARCO^+^ macrophages belong to the resident KCs, while the CD68^+^ MARCO^−^ ones are pro-inflammatory MdMs. In another scRNA-seq study on healthy and cirrhotic human liver samples, the macrophages could be further divided into seven different populations, i.e., three subgroups of tissue monocytes, two of scar-associated macrophages, and two of KCs [11]. Consistently, the fibrotic livers have more scar-associated macrophages, while the healthy livers have more KCs. Due to the sample availability, there are many scRNA-seq studies on liver macrophages in mouse models. The KCs have a high expression of *Adgre1* (encoding F4/80), *Clec4f*, and *Timd4*, as well as a low expression of *Itgam* (encoding Cd11b) [12,13,14,15,16]. The embryo-derived KCs can be further divided into a major population (Cd206^low^ Esam^−^) and a minor population (Cd206^+^ Esam^+^) [17,18]. The recruited MdMs include Clec4f^+^ Tim4^−^ Clec2^+^ monocyte-derived KCs and Clec4f^+^ Tim4^−^ Clec2^−^ MdMs [12,19].

Rats are widely used animal models for pharmacology and toxicology [20]. However, some immune cell markers in rats are different from those in mice. Moreover, many antibodies are not available in rats compared with mice and humans. These limit the use of rat models in MASH studies, which can be overcome by the scRNA-seq technology. MASH animal models are usually generated by gene mutations and/or dietary induction. To study MASH progression with normal dietary, we decided to use our *Leptin* deficient (*Lep^∆I14/∆I14^*) rats [21]. Unlike *Leptin*-deficient *ob/ob* mice [22], which do not develop spontaneous MASH [23,24], *Lep^∆I14/∆I14^* rats have unique MASH phenotypes at postnatal week 16 [25]. Therefore, *Lep^∆I14/∆I14^* rats provide us an excellent platform to investigate the population of macrophages and their diverse functions during MASH progression in the rat liver through scRNA-seq.

## 2. Materials and Methods

### 2.1. Animals

Rats were kept in a 12 h light/12 h dark cycle with ad libitum access to regular chow food and water. The controls were littermates. All the protocols followed the guidelines of the Committee on Animal Care and Use at ShanghaiTech University and Tongji University. Some animal experiments were conducted with the help of Wetry Biotechnology (Shanghai), certified by the Association for Assessment and Accreditation of Laboratory Animal Care (AAALAC). All the experimental procedures were approved by the animal experiment administration committee of Tongji University (no. TJLAC-016-021).

### 2.2. Serum Biochemical Analyses

The serum analyses were performed as previously described [21,25]. Serum alanine aminotransferase (ALT), aspartate aminotransferase (AST), triglyceride, high-density lipoprotein cholesterol (HDL-C), and low-density lipoprotein cholesterol (LDL-C) were determined by automatic biochemistry analyzer (Hitachi 7180, Hitachi High-Tech Corporation, Tokyo, Japan).

Routinely tested blood was measured by automated hematology analyzer (Sysmex XN-1000V, Sysmex Corporation, Kobe, Japan).

### 2.3. Histological Analyses

Liver samples were collected and fixed according to the routine methods for hematoxylin and eosin (HE) staining, Oil Red O staining, Masson’s trichrome staining, and multiplex immunofluorescence (mIF), respectively. For mIF, tissue sections were deparaffinized and subjected to heat treatment using a microwave in pH 9.0 EDTA buffer (Ribibiology, Shanghai, China). Sections were cooled and washed before non-specific binding was blocked. All washing steps were performed 3 times for 5 min in 1× PBS while agitating. After being blocked with 1× PBS with 3% bovine serum albumin (BSA, Solarbio, Beijing, China) for 30 min, the sections were incubated overnight at 4 °C with the primary antibody, followed by incubation with the corresponding secondary antibody. Nuclei were counterstained with 4′,6-diamidino-2-phenylindole (DAPI, Ribibiology, Shanghai, China). The above procedures were repeated multiple times, with each chromogen detecting different antigens based on the specificity of the corresponding primary antibody. The detailed information of the antibodies for mIF was summarized in Table S1.

### 2.4. Generation of Single Cell Suspension

We obtained the rat liver and made the single cell suspension adapted from a previously established protocol [26]. The rats were euthanized. The gastrointestinal organs were moved to the left to reveal the portal vein. The portal vein was snipped before removing a portion of the intact right posterior lobe. After the portal vein was catheterized, the descending vena cava was immediately cut. The liver blanches once the portal vein is catheterized and fully perfuses once the vena cava is cut. Then, the liver was perfused using a pre-warmed (37 °C) perfusion buffer (50 mmol/L EDTA and 10 mmol/L HEPES in 1× HBSS [Gibco, Grand Island, NY, USA]) at an initial flow rate of 3 mL/min for 5 min, and then 4 mL/min for 5 min. The second perfusion was performed with a pre-warmed (37 °C) collagenase type IV (150 U/mL; Invitrogen, Carlsbad, CA, USA) digestion buffer solution (1.25 mmol/L CaCl_2_, 4 mmol/L MgCl_2_, and 10 mmol/L HEPES in 1× HBSS [Gibco]) for 15 min at 4 mL/min. During liver perfusion, the liver was swelled by using forceps to occlude buffer flow from the vena cava every 30 s for 10 s. After surgically removing the gallbladder, the liver was subjected to serial digestions in collagenase type IV (150 U/mL), Accutase (EMD Millipore, Burlington, MA, USA), and trypsin (0.25%) for 30 min, 30 min, and 20 min, respectively, at 37 °C. Dissociated cells were collected after each step and filtered through a 100 μm cell strainer into ice-cold resuspension buffer (1.25 mmol/L CaCl_2_, 4 mmol/L MgCl_2_, 10 mmol/L HEPES, and 5 mmol/L glucose in 1× HBSS supplemented with 2% FBS [Gibco]). The remaining procedure was carried out at 4 °C. Hepatocytes were pelleted by a 30 g spin for 5 min at 4 °C and discarded. The supernatant was centrifuged at 300× *g* for 5 min. The pelleted cells were resuspended in 5 mL red blood cell lysis buffer (BD, San Jose, CA, USA), treated on ice for 10 min, and washed with 10 mL resuspension buffer at 300× *g* for 5 min. The cells were then resuspended in cold resuspension buffer for scRNA-seq.

### 2.5. scRNA-Seq and Bioinformatics

Single cell suspensions (1 × 10^6^/mL) were submitted to 10x Genomics Chromium Controller to generate single-cell gel beads in emulsion. The libraries were constructed by Chromium Single Cell 3′ Library following the manufacturer’s instructions (10× Genomics Chromium Single Cell 3′ Library & Gel Bead Kit v3, 10× Genomics, Pleasanton, CA, USA) and were then sequenced by the Illumina Hiseq 4000 platform.

Raw sequencing data were processed and mapped to the rat genome reference (Rattus_norvegicus.Rnor_6.0) using the CellRanger (7.1.0) pipeline. The generated gene–barcode matrixes were submitted to Scrublet 21 to remove potential doublets. Data filtration, sample integration, gene normalization, dimension reduction, and data visualization were performed after the matrixes were imported into Seurat V3.0 R package (https://satijalab.org/seurat, accessed on 28 December 2024). All the samples were integrated as one object by Seurat IntegrateData function. We excluded cells that exhibited fewer than 300 detected genes and genes expressed in fewer than 3 cells, as well as those with mitochondrial gene representation surpassing 10%. To mitigate potential batch effects arising from sample identity that may disrupt subsequent analyses, we employed the “Harmony” R package (version 0.1.0) for the purpose of batch correction. Dimension reduction was carried out by Seurat RunPCA function. Uniform manifold approximation and projection for dimension reduction (UMAP) was used to visualize single-cell clusters, by graph-based clustering the cells, employing the top 10 principle components with the largest variance (at a resolution of 0.1 for all the merged samples and at a resolution of 0.1 for subclustering macrophages). Manual annotation was carried out for cell identification. The trajectory analyses aimed at forecasting the differentiation pathways of macrophage cell subtypes were conducted utilizing Monocle 2. CellChat was used to infer cell–cell interactions.

Based on the cell clustering results, likelihood ration statistic test was used to screen differentially expressed genes (DEGs) of each cluster by Seurat’s Bimod. The genes can be considered DEGs when they have an expression that satisfies adjusted *p* < 0.05 (corrected *p* value from the Benjamini–Hochberg correction *t*-test) and has a greater than 1.5-fold change (log2 fold change ≥ 0.585) compared to other clusters. Cluster-specific marker genes were chosen by their significant up-regulation in one cluster but not in other clusters.

The rat scRNA-seq data were deposited in the Genome Sequence Archive (GSA) of China National Center for Bioinformation at Beijing Institute of Genomics, Chinese Academy of Sciences. The access number is PRJCA031925.

### 2.6. Statistical Analyses

The data from *Lep^∆I14/∆I14^* mutant rats were compared to the controls by *t*-test. A *p* value of <0.05 was set as significant. The graphs and analyses were performed using GraphPad Prism 6 (GraphPad Software Inc., La Jolla, CA, USA).

## 3. Results

### 3.1. Lep^∆I14/∆I14^ Rats Develop MASH at the Age of Postnatal Week 16

**Figure 1:**
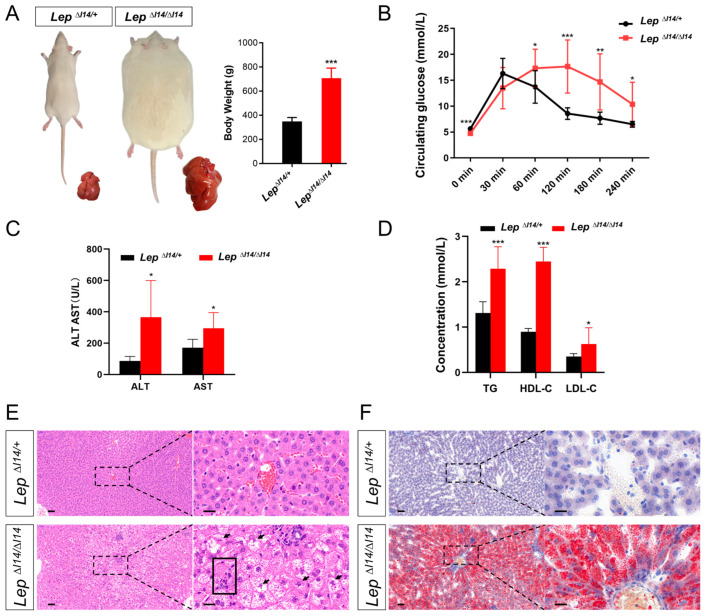
Characterization of MASH phenotypes in *Lep^∆I14/∆I14^* rats at postnatal week 16. (**A**) The left panel shows the representative images of the bodies and livers of an obese male *Lep^∆I14/∆I14^* rat and its litter-mate male control (*Lep^∆I14/+^*), respectively. The right panel shows the body weight statistics (*n* = 6). (**B**) Glucose tolerance experiment was performed by ip injection of male *Lep^∆I14/∆I14^* rats (*n* = 11) and *Lep^∆I14/+^* rats (*n* = 9) with D-glucose. Serum glucose levels were determined at 0, 30, 60, 120, 180, and 240 min after administration. (**C**,**D**) Serum parameters such as ALT and AST (**C**), triglyceride (TG), and HDL-C and LDL-C levels (**D**) in *Lep^∆I14/∆I14^* rats (*n* = 6) and the heterozygous controls (*n* = 6). (**E**) The representative images of the HE staining of liver sections indicate hepatocyte ballooning (arrows) and immune infiltration (solid rectangle) (magnified views in the right panels) in *Lep^∆I14/∆I14^* rats (*n* = 3) compared to the controls (*n* = 3). (**F**) The representative images of Oil Red O staining of liver sections show steatosis in *Lep^∆I14/∆I14^* rats (*n* = 3) compared to the controls (*n* = 3). * *p* < 0.05, ** *p* < 0.01, *** *p* < 0.001. ALT, alanine aminotransferase; AST, aspartate aminotransferase; TG, triglyceride; HDL-C, high-density lipoprotein cholesterol; LDL-C, low-density lipoprotein cholesterol. In (**E**,**F**), the scale bar is 50 µm for the low magnification and 20 µm for the high magnification.

Our previous work showed that *Lep^∆I14/∆I14^* rats start to exhibit MAFLD at postnatal week 8 and develop MASH after week 16 [21,25]. In the present work, we decided to first verify MASH phenotypes in *Lep^∆I14/∆I14^* rats at postnatal week 16. *Lep^∆I14/∆I14^* rats were significantly heavier than their littermate controls (Figure 1A). In the glucose tolerance experiment, after an intraperitoneal injection of D-glucose, we recorded blood glucose concentration at 0, 30, 60, 120, 180, and 240 min, respectively. As shown in Figure 1B, we observed that blood glucose levels in *Lep^∆I14/∆I14^* rats quickly climbed to a peak at 60 min (17.33 ± 3.67 vs. 13.71 ± 3.14 mmol/L in the heterozygous *Lep^∆I14/+^* controls, mean ± SD) and reached a plateau at 120 min (17.66 ± 5.13 vs. 8.57 ± 1.11 mmol/L in the controls, mean ± SD). After 240 min, the blood glucose levels in *Lep^∆I14/∆I14^* rats were still significantly higher than those in the controls (10.36 ± 4.26 vs. 6.50 ± 0.54 mmol/L, Mean ± SD). The complete blood count (CBC) showed that *Lep^∆I14/∆I14^* rats had a significantly reduced number of red blood cells with lower levels of hemoglobin and hematocrit, while they had more white blood cells than the controls (Table S2). Consistently, their ALT and AST levels were significantly higher than those of the controls (Figure 1C). We also observed an increase in serum triglyceride, HDL-C, and LDL-C (Figure 1D). Interestingly, the HDL-C/LDL-C ratio was significantly higher in the mutants than in the controls (4.63 ± 0.55 vs. 2.59 ± 0.16, *p* < 0.01), suggesting that there may be a protective mechanism against cardiovascular diseases in the mutant rats during fatty acid accumulation [27,28,29]. In HE staining, we found MASH phenotypes in *Lep^∆I14/∆I14^* rats, such as steatosis, lymphocyte infiltration, and ballooning (Figure 1E). During Oil Red O staining, we observed increased lipid deposits (Figure 1F). However, we did not see fibrosis in *Lep^∆I14/∆I14^* rats, as assayed by Masson’s trichrome staining (Figure S1). Taken together, our results verified that *Lep^∆I14/∆I14^* rats develop the MASH phenotypes at postnatal week 16.

### 3.2. The Single-Cell Transcriptome of Liver Samples in Lep^∆I14/∆I14^ Rats Reveals Dynamic Proportional Alterations of Different Cell Types in MASH

**Figure 2:**
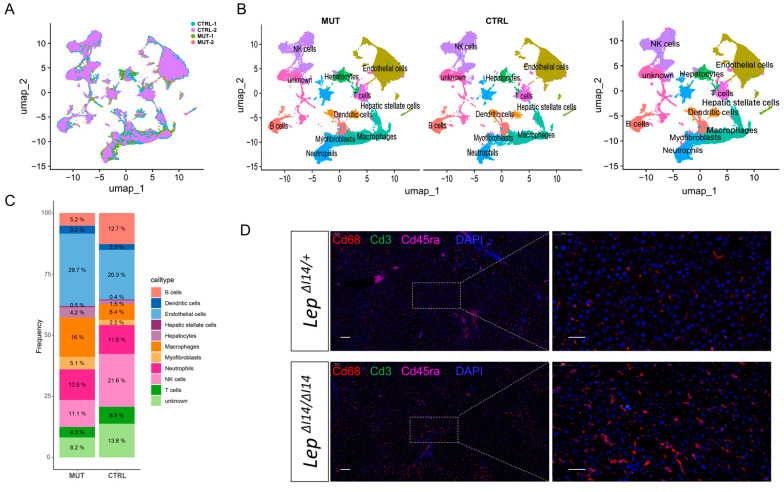
The scRNA-seq showing cellular heterogeneity of liver samples in *Lep^∆I14/∆I14^* rats. (**A**) UMAP plot of the single cells in liver tissues from two male homozygous *Lep^∆I14/∆I14^* rats and two male heterozygous litter mates (*Lep^∆I14/+^*) at postnatal week 16. Different colors labeled for different samples, respectively. (**B**) UMAP plot with different colors labeling different cell types. (**C**) Composition of different cell types in *Lep^∆I14/∆I14^* (MUT) and *Lep^∆I14/+^* (CTRL) rats. (**D**) A representative multiplex immunofluorescence image showing the expression of macrophages (Cd68, red) and T (Cd3, green) and B (Cd45ra, magenta) cells in the liver tissues of *Lep^∆I14/∆I14^* and *Lep^∆I14/+^* rats. The scale bar is 100 µm for the low magnification and 50 µm for the high magnification.

After the assessments described above, we collected liver tissues from two male homozygous *Lep^∆I14/∆I14^* rats with two male heterozygous litter mates (*Lep^∆I14/+^*) as controls. The tissues were immediately processed into single-cell suspensions of non-parenchymal cells before the scRNA-seq libraries were constructed following the manufacturer’s standard protocols (Section 2). There were a total of 1078 Mb reads from 91,193 cells in which a median number of 1668 genes per cell were detected. After filtering out the doublets and cells with poor quality (too few transcripts or too much mitochondria-derived RNA, Figure S2), we obtained 88,758 cells for subsequent analysis. Through the unsupervised clustering visualized by UMAP, we retrieved 32 distinct clusters of the single cells from liver tissues of the mutant and control rats (Figure 2A and Figure S3A). We then analyzed the differentially expressed genes (DEGs) (Figure S3B) and the expression of the canonical markers (Table S3) in each cluster. We grouped the clusters into different cell types such as hepatocytes, hepatic stellate cells, endothelial cells, myofibroblasts, macrophages, NK cells, T cells, B cells, neutrophils, and dendritic cells (Figure 2B). As expected, there were not many hepatocytes (Figure 2C) as they were eliminated by centrifugation during the preparation of the single cell suspension. For the non-parenchymal cells, there were more macrophages and endothelial cells in *Lep^∆I14/∆I14^* rats than in the controls (Figure 2C). However, there was a reduction in the proportions of NK cells and B cells (Figure 2C). To validate the bioinformatic analysis, we performed mIF to visualize the expression location and level of hepatic macrophages. In the healthy controls (Figure 2D), the macrophages stained with anti-Cd68 antibody were evenly distributed. In *Lep^∆I14/∆I14^* rats, however, they tended to cluster together around the pathological steatosis with infiltrated T (stained with anti-Cd3 antibody) and B cells (stained with anti-Cd45ra antibody) (Figure 2D), suggesting the development of inflammation in MASH. Compared to T cells, there were more contacts between the macrophages and B cells, which was consistent with a previous report [30]. Taken together, our results revealed the proportional alterations of different cell types such as macrophages, NK cells, and B cells within the hepatic microenvironment during MASH in *Lep^∆I14/∆I14^* rats.

### 3.3. Hepatic Macrophages Showed Distinct Diversity and Polarization During MASH in Lep^∆I14/∆I14^ Rats

**Figure 3:**
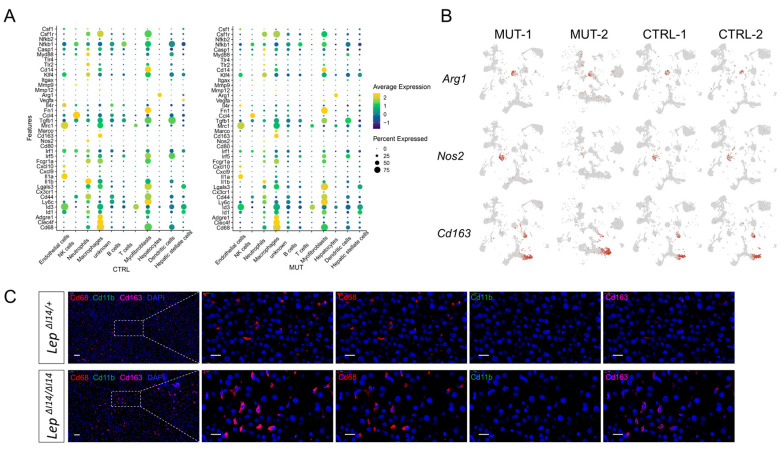
Hepatic macrophages polarization during MASH in *Lep^∆I14/∆I14^* rats. (**A**) DotPlot of expression levels of M1 and M2 markers in different cell types of *Lep^∆I14/∆I14^* (MUT) and *Lep^∆I14/+^* (CTRL) rats. (**B**) UMAP showing the expression of M1 and M2 feature genes, such as *Arg1*, *Nos2*, and *Cd163* in all cell types. (**C**) A representative multiplex immunofluorescence image showing the expression of Cd68 (red), Cd11b (green), and Cd163 (magenta) cells in the liver tissues of *Lep^∆I14/∆I14^* and *Lep^∆I14/+^* rats. The scale bar is 50 µm for the low magnification and 20 µm for the high magnification.

Inflammation in MASH is mostly characterized by immune infiltration, in which macrophages play important roles. As stated above, macrophages account for one of the most abundant populations of non-parenchymal cells in MASH (Figure 2C). Therefore, we decided to investigate the features of hepatic macrophages in *Lep^∆I14/∆I14^* rats. Previous studies showed that hepatic macrophages in MASH were polarized into anti-inflammatory M2 phenotypes. So, we asked if we could identify the macrophage polarization at single cell resolution using the common signature markers of the classic activated macrophage (M1) and alternative activated macrophage (M2) (Table S4). We did not observe a clear expression pattern of macrophage transition from M1 to M2 (Figure 3A). For example, the commonly used M1 marker *Nos2* and M2 marker *Arg1* had very low expression level in both *Lep^∆I14/∆I14^* rats and the controls (Figure 3B). However, the expression level of another M2 marker *Cd163* was high in hepatic macrophages (Figure 3B). In addition, the transcriptional intensity of *Cd163* was higher in *Lep^∆I14/∆I14^* rats than the controls (Figure 3B), suggesting the M1 to M2 transition of macrophages in MASH. To verify this finding at the protein level, we performed mIF using anti-Cd163 antibody (Figure 3C). The anti-Cd68 antibody labeled macrophages in *Lep^∆I14/∆I14^* rats and the controls. As previously reported in human and mouse models, the expression level of Cd11b was low. In line with the scRNA-seq results, the signals of M2 marker Cd163 were increased in the liver of *Lep^∆I14/∆I14^* rats (Figure 3C), supporting the transition of hepatic macrophages to M2 status in MASH.

### 3.4. Hepatic Macrophages Evolved to Distinct Subtypes and Communicated with Other Cell Types in MASH

**Figure 4:**
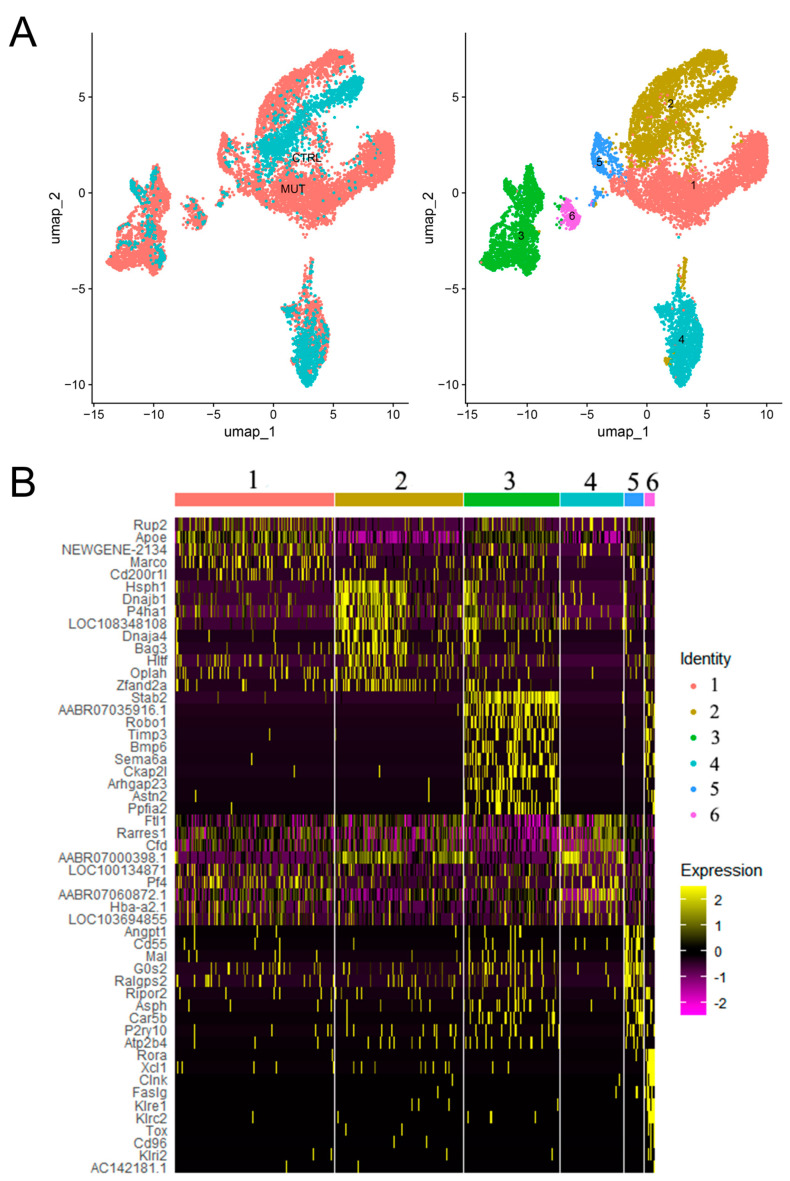
Distinct clusters of hepatic macrophages during MASH in *Lep^∆I14/∆I14^* rats. (**A**) UMAP plot of macrophages with color coded by different tissue origins (**left**). UMAP plot of macrophages with color coded by six clusters (1–6, **right**). (**B**) Heatmap showing the expression of top DEGs in each macrophage cluster.

**Figure 5:**
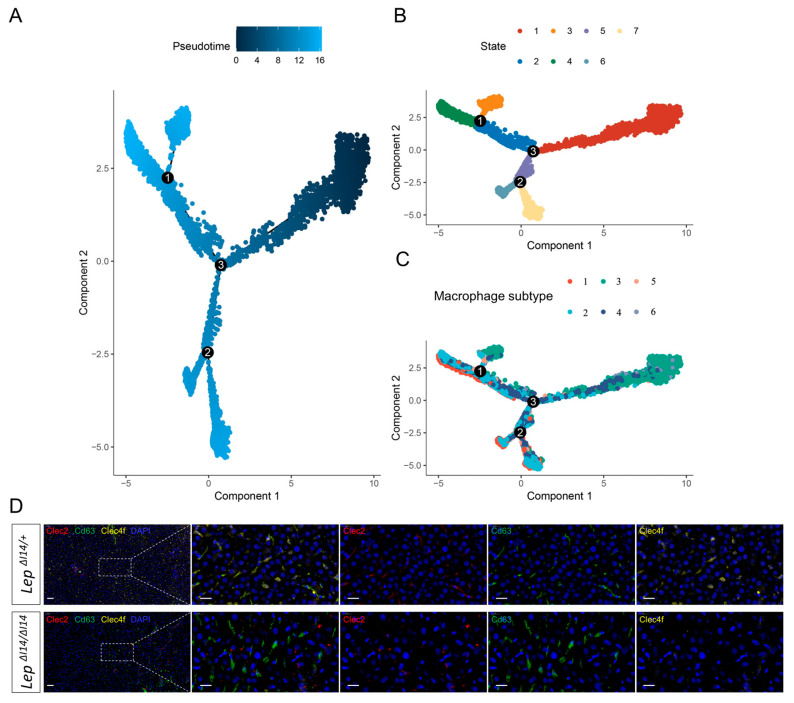
Pseudotime trajectory analysis of the diversity and transition of rat hepatic macrophages in MASH. (**A**) Pseudotime trajectory of all the macrophages colored by their assigned pseudotime values. (**B**) Pseudotime trajectory of all the macrophages with different colors indicating different clusters identified by Seurat. (**C**) Pseudotime trajectory of all the macrophages with different colors indicating different subtypes in Figure 4A. Each dot in the diagram represents a cell, and the numbers in the black circle represent nodes that determine different cell states in the trajectory analysis. (**D**) A representative multiplex immunofluorescence image showing the expression of Clec2 (red), Cd63 (green), and Clec4f (yellow) cells in the liver tissues of *Lep^∆I14/∆I14^* and *Lep^∆I14/+^* rats. The scale bar is 50 µm for the low magnification and 20 µm for the high magnification.

We next sought to explore the diversity of hepatic macrophages during MASH in *Lep^∆I14/∆I14^* rats. Through unsupervised clustering, we obtained six clusters of macrophages (Figure 4A). We found that cluster 1 with high expression of Marco and Apoe was enriched in *Lep^∆I14/∆I14^* rats, while cluster 4 was enriched in the controls (Figure 4B). As Apoe was reported to be a marker of mature lipid associated macrophages (LAMs) and Marco was a M2 marker, our results suggested that rat hepatic macrophages in MASH transited into LAMs with M2 polarization. We then performed a pseudotime trajectory analysis to visualize the diversity and transition of these subtypes during MASH progression (Figure 5A). Overall, hepatic macrophages showed a diverse transition to different directions (Figure 5B). When the six clusters (Figure 4A) were assigned to the pseudotime trajectory, cluster 1 (MASH specific) and cluster 4 (Control specific) did not show an exclusive pattern along the trajectory (Figure 5C). This may imply that the macrophages within each cluster were highly diverse and not committed to a single direction of transition.

Then, we wondered if we could use previous validated markers in mouse models to investigate the transitions of hepatic macrophages in MASH. Clec2 was referred to as an early marker of Mo-KCs, which is highly expressed in most hepatic macrophages except for a small subpopulation of MdMs (Ccr2 and Cx3cr1). Clec4f and Vsig4 largely identify KCs in mouse [31]. Gpnmb or Cd63 are markers for LAMs. In rats, the Clec2 expression was very low (Figure 5D). Most of Cd63 signals, as well as some Clec4f, overlapped with Clec2 in the controls. However, there was an increase in number and intensity of Cd63 signals in *Lep^∆I14/∆I14^* rats, which implied an increase in LAMs in MASH. Meanwhile, the Clec4f signals almost disappeared, suggesting the significant reduction in KCs in MASH (Figure 5D). It should be noted that Clec2 and Cd63 signals did not overlap in *Lep^∆I14/∆I14^* rats (Figure 5D). Nevertheless, LAMs could be identified in the rat MASH model using similar approaches to mouse models.

**Figure 6:**
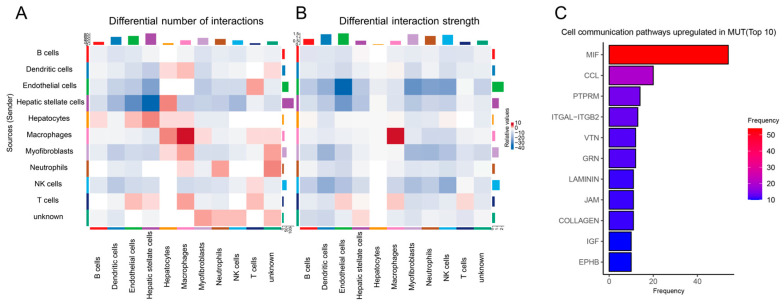
Cell communication among macrophages and other cell types in the hepatic microenvironment during MASH. (**A**) Heatmap showing the differential number of interactions among different cell types between *Lep^∆I14/∆I14^* and *Lep^∆I14/+^* rats. (**B**) Heatmap showing the differential interaction strength among different cell types between *Lep^∆I14/∆I14^* and *Lep^∆I14/+^* rats. (**C**) Top 10 cell communication pathways upregulated in *Lep^∆I14/∆I14^* rats (MUT). MIF, macrophage migration inhibitory factor; CCL, chemokine (C-C motif) ligand; PTPRM, protein tyrosine phosphatase receptor type M; ITGAL-ITGB2, integrin subunit alpha L-integrin subunit beta 2; VTN, vitronectin; GRN, granulin precursor; JAM, F11 receptor; IGF, insulin-like growth factor; EPHB, ephrin receptor.

As macrophages interact with other cell types in the hepatic microenvironment, we decided to explore their communication with other cell types in MASH. We first examined the number of interactions among different cell types in *Lep^∆I14/∆I14^* rats and the controls (Figure S4A,B). We found that macrophages had increased the differential number of interactions with hepatocytes, myofibroblasts, T cells, neutrophils, and dendritic cells (Figure 6A). We did not observe differences in the incoming and outgoing interaction strengths of macrophages in MASH (Figure S4C,D). However, we found an increased interaction strength between macrophages and T cells (Figure 6B). We did not observe an increased number and strength of interactions between macrophages and B cells. Finally, we analyzed the differential cell communication pathways between *Lep^∆I14/∆I14^* rats and the controls. We found that the macrophage migration inhibitory factor (MIF) pathway was highly enriched in the liver of *Lep^∆I14/∆I14^* rats (Figure 6C), suggesting that it may facilitate macrophages to communicate their microenvironment and regulate MASH progression.

## 4. Discussion

To the best of our knowledge, it is the first time that hepatic macrophages, key players in inflammation, have been investigated at the single cell resolution in a rat MASH model. Taking advantage of our previously characterized *Lep^∆I14/∆I14^* rats, which developed MASH phenotypes after postnatal week 16, we discovered the characteristics of macrophage subtypes and polarization during MASH progression. We validated Cd163 and Cd63 markers as M2 and LAM markers in rats, respectively. Moreover, we identified the cell–cell interactions between macrophages and other cell types, in which the top upregulated MIF pathway may play important roles in MASH. In conclusion, our results revealed macrophage dynamics during MASH at single cell resolution in rats.

Macrophage polarization is always discussed in many inflammation-related diseases such as MASH and cancer. There are different terminologies which are debated in the field [32]. The widely used terms M1 and M2 were proposed when Mills et al. studied the different metabolisms of arginine in T cells (T helper 1 (Th1) and Th2) and macrophages (M1 and M2) in C57BL/6 and BALB/c mice [33]. The predominant Th1 cell and M1 responses in C57BL/6 mice were correlated with a deletion in the promoter of *Slc7a2* which encodes the arginine transporter in macrophages [34]. Compared to M2-prone BALB/c mice, a methionine–choline-deficient (MCD) diet in M1-prone C57BL/6 mice had high chance of resulting in liver steatosis and inflammation [35]. M1 macrophages were increased during high-fat-diet (HFD) feeding in mice, while M2 polarization could partially reversed the MAFLD phenotypes [36]. It seems that M2 macrophages were associated with improved insulin sensitivity and reduced steatosis in MAFLD [36,37,38]. However, there was much controversial evidence. For example, the expression M2 markers were elevated in MASH patients, suggesting they might be involved in promoting tissue repair during MASH progression [39]. In MCD diet-fed mice, M1 macrophages peaked at 4 weeks and decreased thereafter [40]. M1 mice generated by IL-10/IL-4 deletion were resistant to MASH, while M2 mice generated by IFN-γ deletion quickly developed MASH [41]. M1 macrophages with pro-inflammatory markers were not observed in obesity and insulin resistance in mice and humans [42]. Most of the aforementioned studies used either human or mouse samples. Previously, we used M1 and M2 markers to investigate hepatic macrophages in *Lep^∆I14/∆I14^* rats [25]. However, our experiments were limited by the available antibodies for rats. In this work, we addressed this problem by using scRNA-seq. We found that hepatic macrophages in rats underwent a unique M1 to M2 transition without a distinct pattern at transcription level. For example, the expression of the classical markers such as Arg1 and Nos2 is very low in both *Lep^∆I14/∆I14^* rats and the controls (Figure 3). However, Cd163, a well-defined M2 marker [43] was confirmed to be highly expressed in the hepatic macrophages of *Lep^∆I14/∆I14^* rats at RNA and protein levels (Figure 3). Taken together, our results support the argument that M1 to M2 transition is a spectrum instead of isolated stages [32].

The markers of liver macrophages are mostly characterized by IHC and cytometry in mouse models. The known markers of murine hepatic macrophages include F4/80 (Adgre1), Cd11b, Timd4 (TIM4), and Vsig4. Generally, hepatic macrophages can be identified by F4/80^hi^, CD11b^int^. The expression of Timd4 can distinguish embryonically derived resident KCs and recruited MdMs in the sinusoidal macrophages [44]. F4/80^hi^, CD11b^int^, and Timd4^hi^ macrophages are KCs, while F4/80^hi^, CD11b^int^, and Timd4^lo^ ones are MdMs, which is associated with MASH severity [31]. MdMs can be further divided into Timd4^lo^ Vsig4^hi^ (or CLEC4F^hi^) monocyte-derived KCs (mo-KCs) and Timd4^lo^ Vsig4^lo^ LAMs [31]. LAMs, also known as MASH-associated macrophages (NAMs), are characterized by the expression markers Trem2, Cd9, Cd63, and Gpnmb [16]. Clec2 [45] is highly expressed by most liver macrophages except for a small subpopulation of Ccr2- and Cx3cr1-positive MdMs (C-LAM). The population with a low expression of Clec2 may include liver capsular cells and/or an early precursor of LAMs. There are several known LAM markers such as Trem2, Gpnmb, and Cd63. In the immunofluorescence and confocal imaging, the co-staining of Cd63 or Gpnmb with Clec4f and Ccr2-GFP can be used to distinguish LAMs, KCs/mo-KCs and C-LAMs, respectively [46]. However, the markers of hepatic macrophages, especially antibodies for immunofluorescence, were not well characterized in rats. In this work, we performed mIF to validate Cd68, Cd3, and Cd45ra for hepatic macrophages, T and B cells in rats, respectively. Moreover, we validated the M2 marker Cd163 and the LAM marker Cd63. Thus, our work offers invaluable tools to study the diversity and function of hepatic macrophages in rats.

MIF is a pro-inflammatory cytokine with chemokine-like functions. It is released from immune cells such as macrophages modulates inflammatory kinase pathways, PI3K/AKT- and p53-mediated macrophage survival, and leukocyte recruitment [47]. MIF exerts its function of promoting leukocyte recruitment to inflammatory sites by noncognate interaction with the chemokine receptors CXCR2 and CXCR4 [48], and regulates the MIF-stimulated survival of macrophages, B cells, and tumor cells through its receptor cluster of differentiation 74 (CD74) [49]. In metabolic syndromes, MIF is pleiotropic. It promotes ethanol-induced liver injury in patients with alcohol-associated hepatitis [50,51]. However, MIF was a protective factor for high-fat diet-induced liver injury and chemically induced liver fibrosis [52,53,54]. We found that *Lep^∆I14/∆I14^* rats develop MASH without fibrosis (Figure 1 and Figure S1) [25]. Therefore, MIF may play a role in modulating diabetes progression and limiting inflammatory strength. As *Lep* is deficient in the MASH model, Lep may be involved in the upstream and/or downstream regulation of the MIF pathway, which should be further investigated in the future.

MASH is a complex disease whose pathology can be explained through the “multiple hits” theory [55]. In *Lep^∆I14/∆I14^* rats, we observed a stepwise change in hepatic gene expression in the pathways of lipid synthesis, insulin resistance, inflammation, reactive oxygen species, endoplasmic reticulum stress, and mitochondrial function from postnatal weeks 4 to 48, respectively [25]. In the present work, we consistently observed MASH phenotypes in *Lep^∆I14/∆I14^* rats at postnatal week 16. However, the limitation is that we did not recognize the transition point between simple steatosis and MASH when MASH was triggered by a second hit or by multiple hits. Addressing this issue will also help to explain the difference in MASH progression between *Leptin*-deficient rats (*Lep^∆I14/∆I14^*) and mice (*ob/ob*). Therefore, adding more timepoints between week 8 and 16 will be an important focus of our future experiments.

## 5. Conclusions

Here, we revealed hepatic macrophage dynamics during MASH at single cell resolution and provided fundamental tools for the investigation of MASH in rat models. By scRNA-seq of the liver tissues from a previously characterized rat MASH model *Lep^∆I14/∆I14^*, we found an increase in number of macrophages and endothelial cells and a decrease in that of NK and B cells. We discovered that hepatic macrophages in rats had a unique M1 to M2 transition during MASH, and they had an increased number of interactions with hepatocytes, myofibroblasts, T cells, neutrophils, and dendritic cells. Meanwhile, we validated several hepatic cell markers for investigation of MASH in rats. 

## Data Availability

The rat scRNA-seq data are available at GSA with the access number PRJCA031925.

## Supplementary Materials

The following supporting information can be downloaded at https://www.mdpi.com/article/10.3390/cells14020096/s1. Supplementary Figure S1. The representative images of Masson’s trichrome staining of liver sections indicated no fibrosis in *Lep^∆I14/∆I14^* rats compared to control *Lep^∆I14/+^* rats; Supplementary Figure S2. Number of genes, number of transcripts, percentage of mitochondrial genes, percentage of ribosomal genes, and percentage of red blood cell genes before cell filtration for the four scRNA-seq data CTRL-1 (A), CTRL-2 (B), MUT-1 (C), and MUT-2 (D), respectively. Doublets (red) of CTRL-1 (E), CTRL-2 (F), MUT-1 (G), and MUT-2 (H) were excluded in the analysis of Figure 2A–C; Supplementary Figure S3. Differential gene expressions in different cell clusters and cell types; Supplementary Figure S4. Cell–cell interaction analysis; Supplementary Table S1. Antibody information for multiplex immunofluorescence (mIF); Supplementary Table S2. The significantly changed parameters in the complete blood count (CBC) of *Lep^∆I14/∆I14^* (*n* = 9) and *Lep^∆I14/+^* (*n* = 7) rats; Supplementary Table S3. Canonical markers for each cell type; Supplementary Table S4. M1 and M2 signature genes.
