# Supplementary material for: Single-Cell RNA Sequencing Reveals Macrophage Dynamics During MASH in Leptin-Deficient Rats
Source: Cells. 2025 Jan 10;14(2):96. doi: 10.3390/cells14020096 (PMC11763963; doi:10.3390/cells14020096)
Supplement: Supplementary file 1 [file cells-14-00096-s001.zip › Supplementary Figures-250106.pdf]

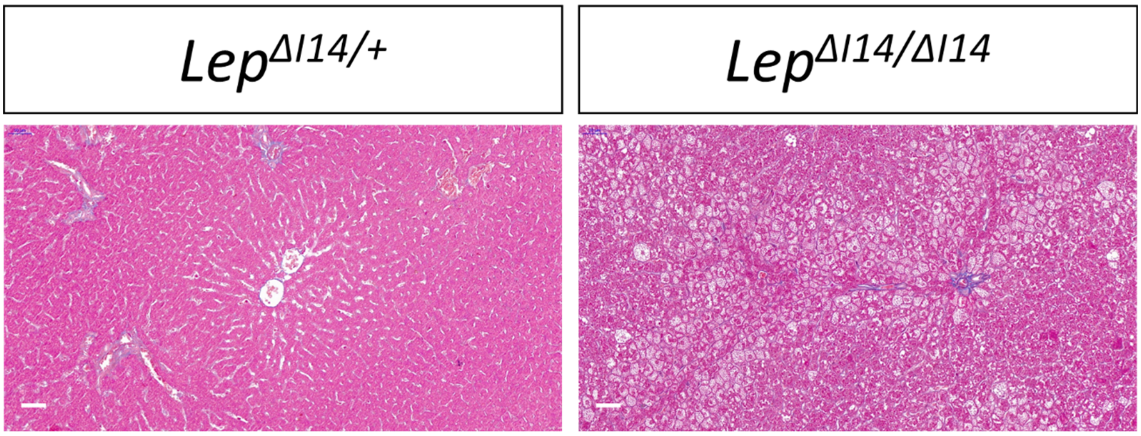

Supplementary Figure S1. The representative images of Masson's trichrome staining of liver sections indicated no fibrosis in in $Lep^{\Delta I14/\Delta I14}$ rats compared to the control $Lep^{\Delta I14/+}$ rats. The scale bar is 50 μm.

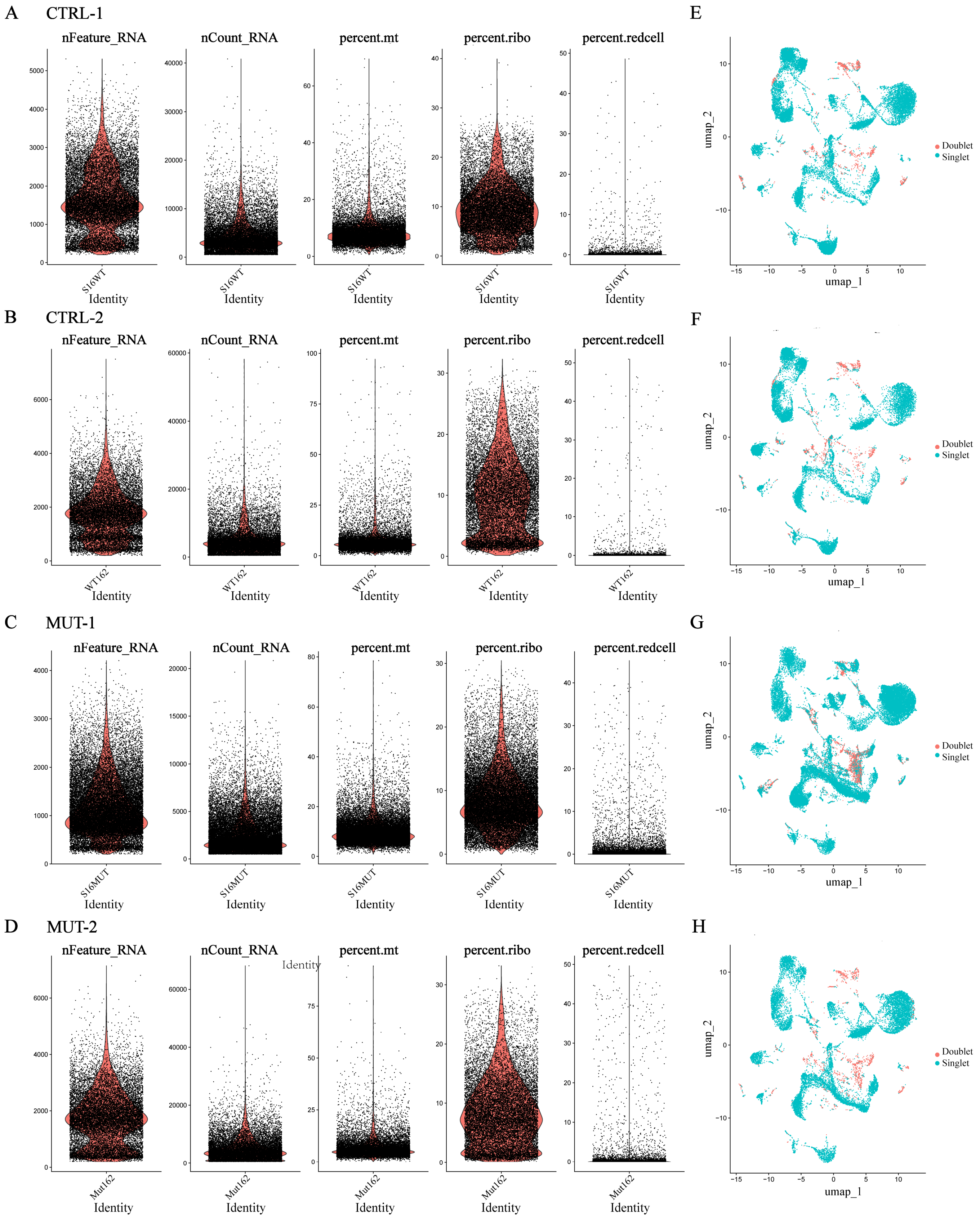

Supplementary Figure S2. Number of genes, number of transcripts, percentage of mitochondrial genes, percentage of ribosomal genes, and percentage of red blood cell genes before cell filtration for the four scRNA-seq data CTRL-1 (A), CTRL-2 (B), MUT-1 (C) and MUT-2 (D), respectively. The quality control

included removing doublets by Scrublet and filtering cells that meet the criteria: 200<number of genes<10000, percentage of mitochondrial genes<10%, and percentage of red blood cell genes<10%. Doublets (red) of CTRL-1 (E), CTRL-2 (F), MUT-1 (G) and MUT-2 (H) were excluded in the analysis of Figure 2A-C.

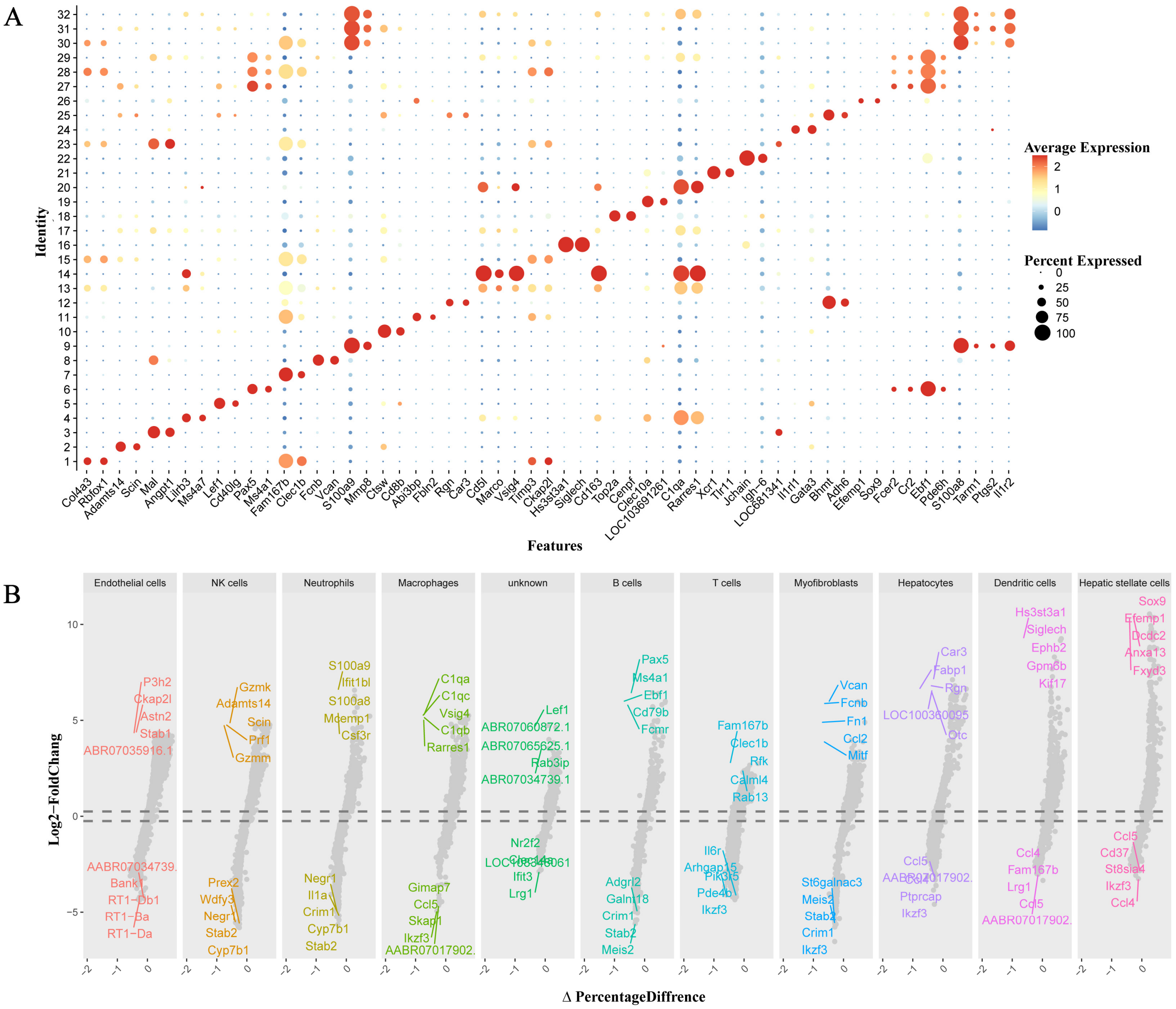

Supplementary Figure S3. Differential gene expression in different cell clusters and cell types. (A) The expression of top DEGs in 32 clusters of the single cells from liver tissues of *Lep* $^{\Delta I14/\Delta I14}$ and *Lep* $^{\Delta I14/+}$ rats. (B) Differential gene expression analysis [log2-fold change expression versus the difference in the percentage of cells expressing the gene (Δ Percentage Difference)] showing top 5 up- and down-regulated genes across all cell types.

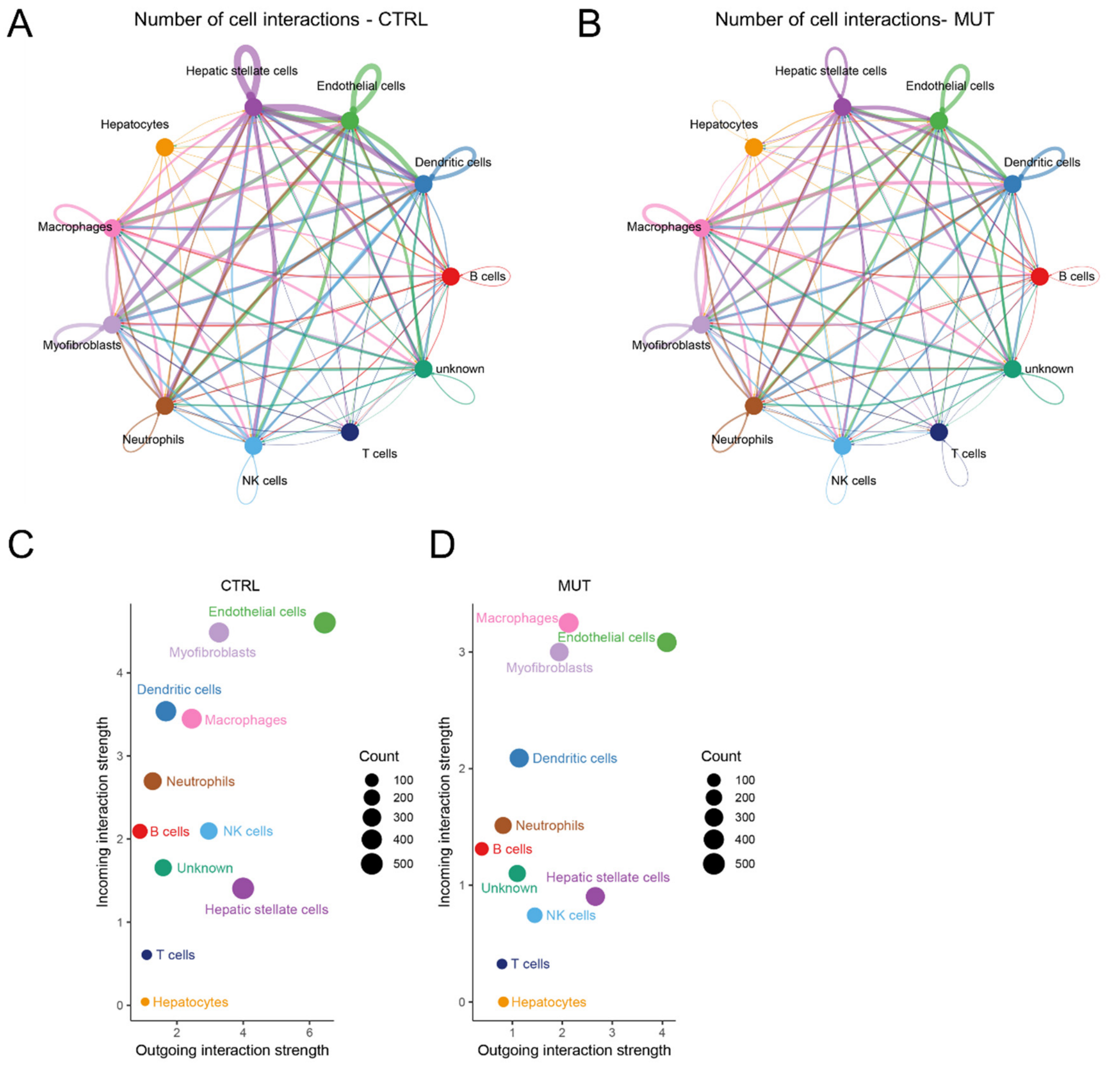

Supplementary Figure S4. Cell-cell interaction analysis. (A) Number of interactions of different cell types in *Lep* $^{\Delta I14/+}$ rats (CTRL) visualized by netVisual_bubble function. (B) Number of interactions of different cell types in *Lep* $^{\Delta I14/\Delta I14}$ rats (MUT) visualized by netVisual_bubble function. (C) The incoming and outgoing interaction strength of different cell types in *Lep* $^{\Delta I14/+}$ rats (CTRL) visualized by netVisual_aggregate. (D) The incoming and outgoing interaction strength of different cell types in *Lep* $^{\Delta I14/\Delta I14}$ rats (MUT) visualized by netVisual_aggregate.
