# Supplementary material for: Single-Cell RNA Sequencing Reveals Macrophage Dynamics During MASH in Leptin-Deficient Rats
Source: Cells. 2025 Jan 10;14(2):96. doi: 10.3390/cells14020096 (PMC11763963; doi:10.3390/cells14020096)
Supplement: Supplementary file 1 [file cells-14-00096-s001.zip › Supplementary Tables.pdf]

Supplementary Table S1. The primary antibody information for multiplex immunofluorescence (mIF).

Target	Host	Vendor	Catalogue number	Dilution
Clec1b (Clec2)	rabbit	Affinity	DF14376	1:200
Cd63	rabbit	Proteintech	67605-1-Ig	1:200
Clec4f	rabbit	Biorbyt	orb512932	1:100
Cd3	rabbit	Abcam	ab318146	1:500
Cd45ra	mouse	Santa Cruz	sc-53048	1:200
Cd68	rabbit	Abcam	ab283654	1:200
Cd11b	rabbit	Abcam	ab133357	1:1000
Cd163	rabbit	Abcam	ab316218	1:200

Supplementary Table S2. The significantly changed parameters in the complete blood count (CBC) of *Lep*$^{\Delta I14/\Delta I14}$ (n=9) and *Lep*$^{\Delta I14/+}$ (n=7) rats.

**Genotype**	**RBC (10^12/L)**	**HGB (g/L)**	**HCT (%)**	**WBC (10^9/L)**
Lep $^{\Delta I14/\Delta I14}$	7.51±0.60*	151.89±8.36*	36.08±2.51**	10.40±0.29*
Lep $^{\Delta I14/+}$	8.13±0.34	163.71±8.71	40.03±1.70	8.24±0.82

* P<0.05, ** P<0.01

RBC, red blood cells; HGB, hemoglobin; HCT, hematocrit; WBC, white blood cells.

Supplementary Table S3. Canonical markers for each cell type.

Cell type	Marker genes
Hepatocytes	*Alb, Krt18, Hnf4a*
Monocytes/ macrophages	*Cd68, Csf1r, Adgre1 (F4/80), Cd14, Cd163, Fcgr3a, Fcgr1a*
T cells	*Cd3d, Cd3e, Cd3g*
Dendritic cells	*Itgax (Cd11c), Il3ra (Cd123), Itgae (Cd103), Ccl17, Ccl22, Cd207*
B cells	*Igh-6 (IgM), Cd19, Ms4a1 (Cd20), Cd79b*
NK cells	*Ncr1 (Nkp46), Klrb1c (Cd161), Klrk1 (Nkg2d)*
Neutrophils	*Cd177, Fut4 (Cd15)*
Stellate cells	*Acta2 (alpha-SMA), Gfap*
Endothelial cells	*Mcam (Cd146), Vcam1 (Cd106)*

Supplementary Table S4. M1 and M2 signature genes.

M1 signature genes	M2 signature genes
Nos2, Il1b, Il1a, Tnf, Lgals3 (Galectin 3), Il6, Cxcl9, Cxcl10, Il12a, IL12b, IL23a, Fcgr1a, Fcgr1B, Fcgr1C, Ccr7, Il8, Ccl5, Irf5, Irf1	*Arg1, Il10, Cd163, Marco, Mrc1, Msr1, Stab1, Tgm2, Mmp7, Mmp9, Mmp12, Mmp13, Mmp19, Tgfb1, Tgfb2, Tgfb3, Vegfa, Fn1, Ccl4, Ccl22, Ccl17, Ccl18, Il4r, Il7r, Irf4*
